# Supplementary material for: Multifunctional 3D-Printed Magnetic Polycaprolactone/Hydroxyapatite Scaffolds for Bone Tissue Engineering
Source: Polymers (Basel). 2021 Nov 5;13(21):3825. doi: 10.3390/polym13213825 (PMC8588077; doi:10.3390/polym13213825)
Supplement: Supplementary file 1 [file polymers-13-03825-s001.zip › Supplementary Figure S1.pdf]

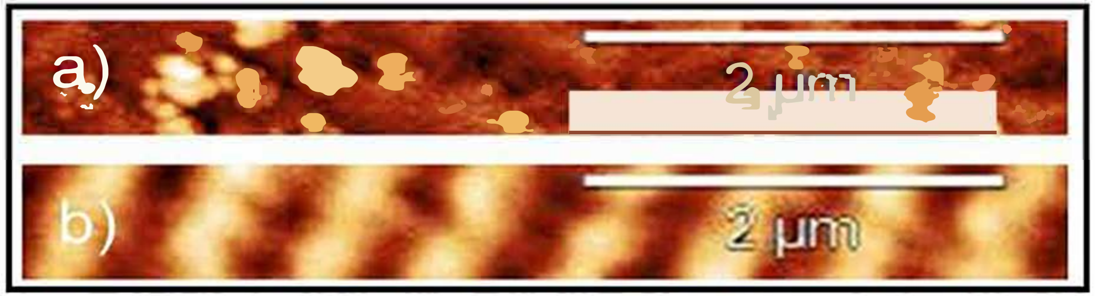

Supplementary Figure S1. Magnetic Force Measurement Calibration. a) topography of the calibration sample and (b) its corresponding magnetic phase signal.

Previous calibration of the magnetic measurement by AFM is performed by using an opportune calibration grid exhibiting magnetic pattern regularly spaced (about 1 um spacing). While topographic signal is acquired, magnetic phase signal in double pass lift mode (see the main text) is also acquired. Patterning recognition in the magnetic phase signal means that the selected measurement parameters (lift distance, gain, et.) are set correctly. In Fig. 1
