# Supplementary material for: Multifunctional 3D-Printed Magnetic Polycaprolactone/Hydroxyapatite Scaffolds for Bone Tissue Engineering
Source: Polymers (Basel). 2021 Nov 5;13(21):3825. doi: 10.3390/polym13213825 (PMC8588077; doi:10.3390/polym13213825)
Supplement: Supplementary file 1 [file polymers-13-03825-s001.zip › Supplementary Figure S2.pdf]

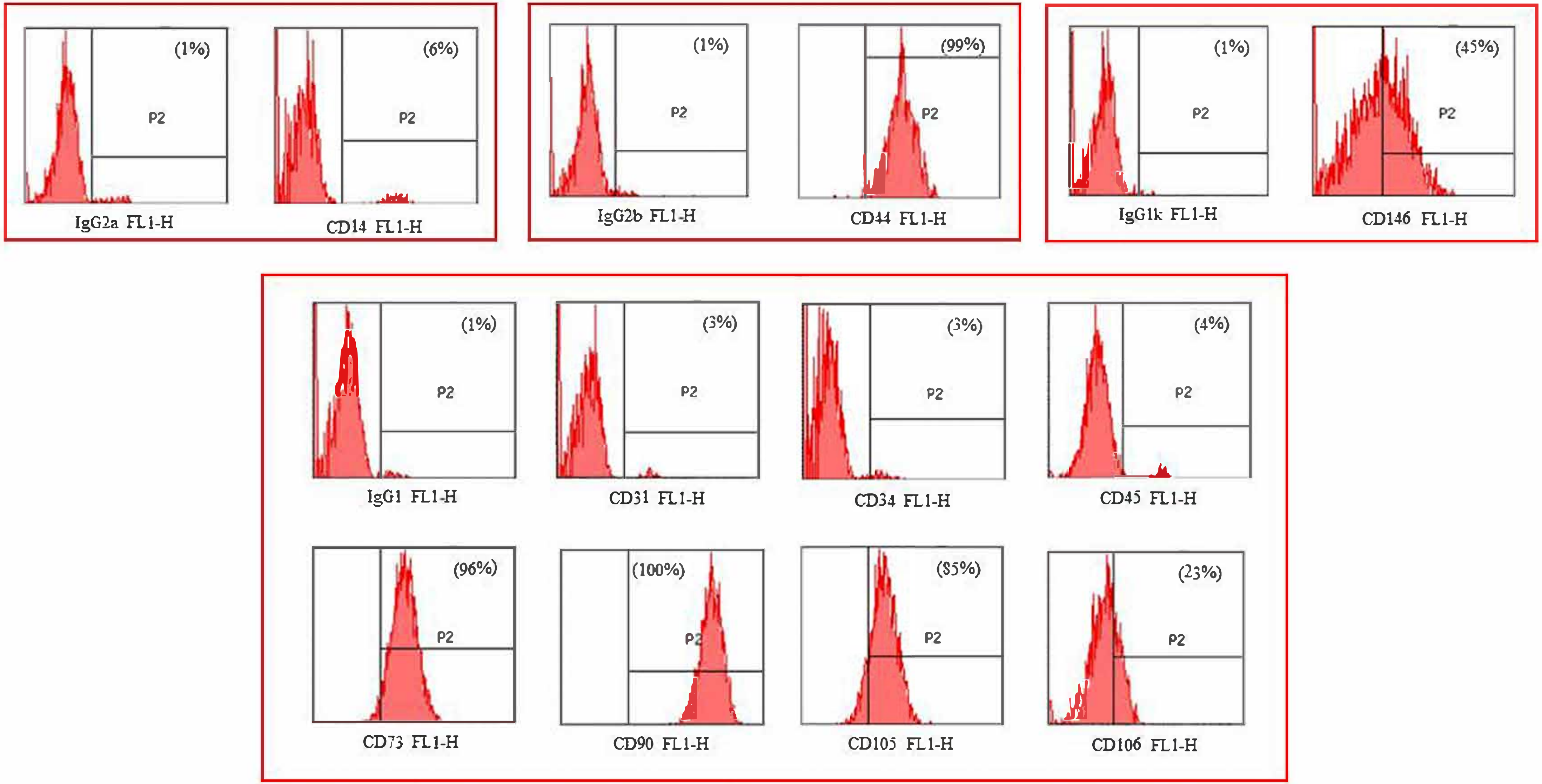

Supplementary figure S2. FACS Analysis of typical hematopoietic and mesenchymal markers in a representative sample of mesenchymal stromal cells (MSCs). Histograms reporting the percentage of positivity (P2) for the following markers: CD-14, CD-44, CD-146, CD31, CD-34, CD-45, CD-73, CD-90, CD-105, CD-106 grouped in rectangular forms with their relative isotype controls.
