# Supplementary material for: Multifunctional 3D-Printed Magnetic Polycaprolactone/Hydroxyapatite Scaffolds for Bone Tissue Engineering
Source: Polymers (Basel). 2021 Nov 5;13(21):3825. doi: 10.3390/polym13213825 (PMC8588077; doi:10.3390/polym13213825)
Supplement: Supplementary file 1 [file polymers-13-03825-s001.zip › Supplementary Figure S3.pdf]

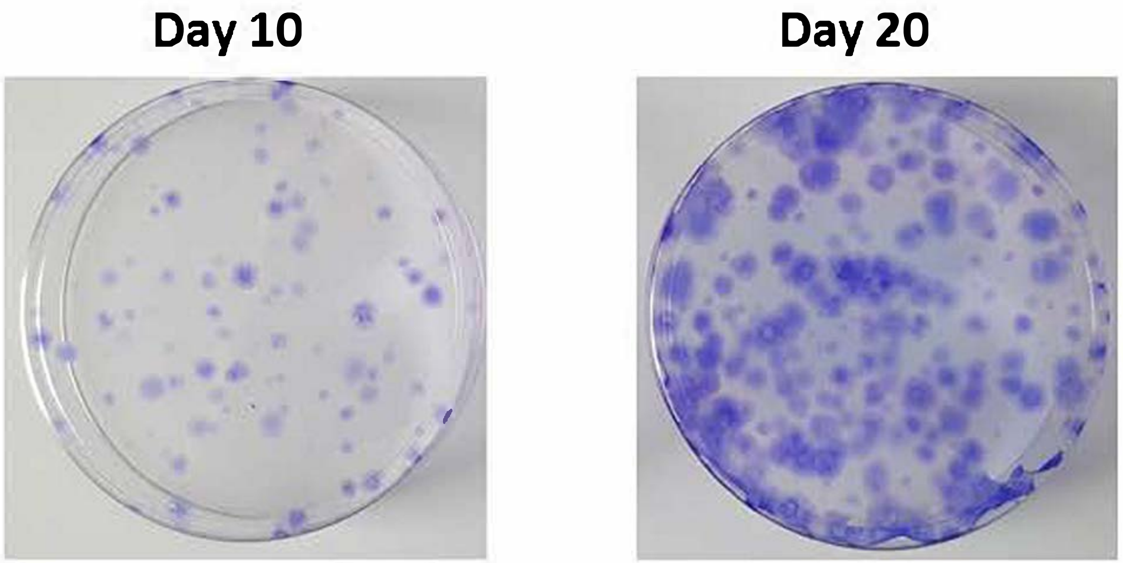

Supplementary Figure S3. CFU-F analysis. Representative images of CFU-F (colony-forming-units fibroblasts) formation of mesenchymal stromal cells stained with Crystal Violet at 10 and 20 days.
