# Supplementary material for: Multifunctional 3D-Printed Magnetic Polycaprolactone/Hydroxyapatite Scaffolds for Bone Tissue Engineering
Source: Polymers (Basel). 2021 Nov 5;13(21):3825. doi: 10.3390/polym13213825 (PMC8588077; doi:10.3390/polym13213825)
Supplement: Supplementary file 1 [file polymers-13-03825-s001.zip › Supplementary Figure S4.pdf]

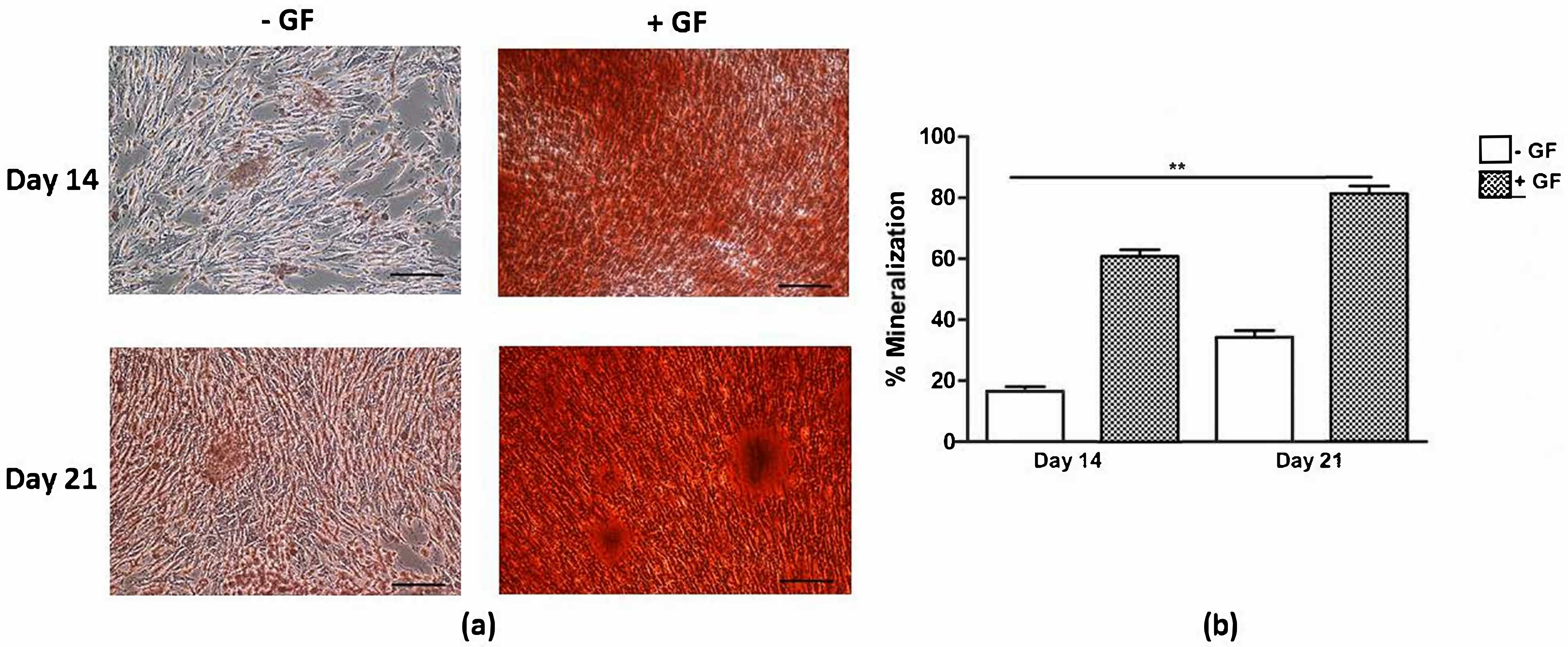

Supplementary figure S4. Osteogenic differentiation of MSCs (**a**) Micrographs of MSCs cultured with (+GF) and without growth factors (-GF) and stained with Alizarin Red S solution at 14 and 21 days. Scale bar: 100 µm; Red staining: mineralization areas. (**b**) Graphical representation of % mineralization. Data are reported as mean ± standard deviation. Kruskall-Wallis and post hoc Dunn's test were used for statistical analysis. **P<0.01 MSCs at day 14 cultured without growth factors (-GF) versus MSCs at day 21 cultured with growth factors (+GF).
